# Comparison of component and quality changes between soft rice and common rice during aging

**DOI:** 10.3389/fnut.2025.1656432

**Published:** 2025-09-10

**Authors:** Yihang Wang, Chunsen Wu, Guodong Liu, Lunan Guo

**Affiliations:** ^1^School of Food Science and Engineering, Yangzhou University, Yangzhou, China; ^2^Jiangsu Key Laboratory of Crop Genetics and Physiology, Agricultural College of Yangzhou University, Yangzhou, China; ^3^Jiangsu Key Laboratory of Crop Cultivation and Physiology, Agricultural College of Yangzhou University, Yangzhou, China; ^4^Jiangsu Co-Innovation Center for Modern Production Technology of Grain Crops, Agricultural College of Yangzhou University, Yangzhou, China

**Keywords:** soft rice, aging, quality, component, structure

## Abstract

**Background:**

Compared to common rice, soft rice is characterized by low amylose content, soft and elastic texture, and low retrogradation of cold rice. However, the differences of quality deteriorate during aging between soft rice and common rice are still unclear.

**Methods:**

In this study, representative soft rice varieties (NJ9108 and NJ46) were chosen as research subjects, and YJ7081 and HD5 were chosen as control. The changes of their components and quality during aging were comprehensively investigated.

**Results:**

During aging, the total starch, fat, and protein content of soft rice decrease, while the amylose content increases. The short-range ordered structure of soft rice starch and the secondary structure of proteins gradually degrade with the extension of aging time. In addition, the relative crystallinity of soft rice starch gradually decreases during the aging process. After aging for 24 M, the average taste value of soft rice decreased by 14.86, and the average 2-AP content decreased by 167.82 ng/g. The average taste value of common rice decreased by 12.52, and the average 2-AP content decreased by 140.42 ng/g.

**Conclusion:**

Compared to common rice, the component contents and the starch short-range ordered structure characteristics of soft rice exhibited more pronounced changes, leading to increased deterioration of cooking, eating, and aroma qualities during aging.

## Graphical abstract

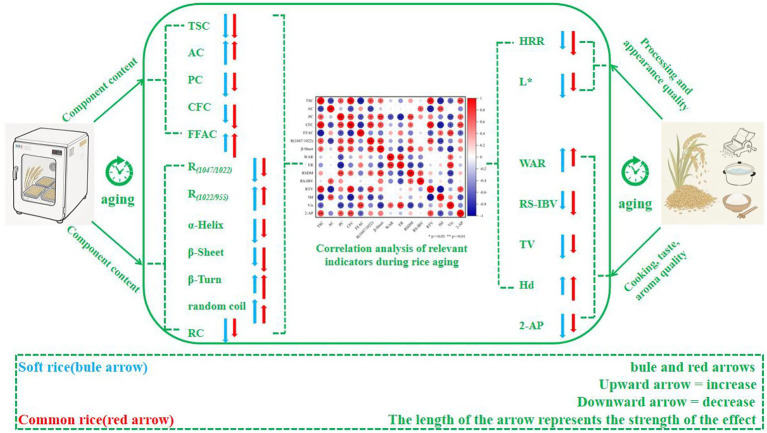

## Highlights

- Soft rice quality declines faster than common rice during aging.
- Structural degradation of starch and protein is more severe in soft rice.
- Aging leads to reduced cooking, eating, and aroma qualities in soft rice.
- Faster loss of 2AP in soft rice contributes to greater aroma quality deterioration.

## 1 Introduction

Rice is among the three major staple crops worldwide serving as a primary food source for over 60% of the population in China (1). Recent development in social economy and improvement in living standards have significantly increased the societal demand for high-quality food. To improve the eating quality of rice, various high-quality soft rice varieties with superior eating characteristics have been identified and cultivated in the southern japonica rice-growing regions of China (2). Soft rice exhibits a relatively low amylose content (AC; 8–13%), which lies between those of glutinous and regular rice. It is also referred to as semi-glutinous japonica rice (3). Soft rice exhibits a tender but firm texture when cooked, whereas cold rice exhibits low retrogradation, which is highly preferred by consumers. Over the past two decades, studies have extensively investigated the agronomic traits associated with the formation, processing, appearance, and cooking and eating qualities of soft rice (4), leading to the development of new soft rice varieties and enhancing the overall quality of japonica rice in southern China (5).

Physicochemical properties of rice change during the storage period before sale. This process is known as aging (6). In recent years, the number of studies has been carried out on the quality changes of rice during aging. Most studies attributed the changes during rice aging to changes in cell wall strength, chemical composition (starch, protein, lipids, free fatty acids and their interactions) (7), and endogenous enzymatic reactions (8). For example, due to the action of enzymes, the amylose content increases during rice aging, resulting in a decrease in the viscosity of rice (9). In addition, during the aging, the starch in rice will regenerate (essentially the starch recrystallization process), which is the main reason for the deterioration of rice quality during storage (10). In addition, due to the interference of biological factors such as insects and microorganisms during storage, the quality of rice will be further reduced (11).

With the advancement of plant breeding technology, soft rice varieties with relatively low AC (5–15%) have been developed in southern China, among which Nanjin46 and “Nanjin9108” have been highly rated in the China-Japan Rice Tasting Competition for many times. As a result, breeders have paid more attention to soft rice varieties, and many cultivars are gradually being planted in a large area in China’s Yangtze River Delta (12). However, the amylose content in soft rice is low, and the internal structure of starch tends to form a loose, porous structure, which will affect the hardness of rice grains. Additionally, the starch granules in the endosperm of soft rice are arranged loosely, with numerous cavities between them, allowing substances such as water and oxygen to interact more easily with the external environment, further impacting the quality of the soft rice (3). Moreover, specific changes during aging, particularly the distinction between soft and common rice varieties, remain unknown. Therefore, this study used two representative soft *Japonica* rice varieties [Nanjing9108 (NJ9108) and Nanjing46 (NJ46)] as research subjects and common *Japonica* rice varieties are widely distributed in southern China (2) [Yangjing 7081 (YJ7081) and HuaiDao 5 (HD5)] as controls to systematically examine the changes in rice component and quality changes during aging. The samples were provided by the College of Agriculture, Yangzhou University, China. This study explored to provide a theoretical foundation for the breeding and storage of high-quality soft rice varieties.

## 2 Materials and methods

### 2.1 Test materials

Soft *Japonica* (NJ9108 and NJ46) and common *Japonica* (YJ7081 and HD5) rice varieties were cultivated in an experimental field at Yangzhou University under standardized growing conditions from May to November, 2022. Rice variety growth period: NJ9108 (152 days); NJ46 (165 days); YJ7081 (152 days); HD5 (152 days).

The soil in the experimental field was sandy loam, containing 0.13% total nitrogen, 87.3 mg·kg^−1^ alkali-hydrolyzable nitrogen, 32.5 mg·kg^−1^ available phosphorus and 88.5 mg·kg^−1^ available potassium. A randomized block design was adopted, with row spacing of 30 cm × 12 cm and 4 seedlings per hole. The plot area is 36 m^2^. The nitrogen fertilizer application rate was 270 kg·hm^−2^, and the nitrogen fertilizer was applied according to the ratio of base tillering fertilizer to panicle fertilizer = 7:3. Base fertilizer: tillering fertilizer: panicle fertilizer = 3.5:3.5:3.0. Tillering fertilizer was applied 7 days after transplanting, and panicle fertilizer was applied at the fourth leaf stage. The ratio of nitrogen (pure N): phosphorus (P_5_O_2_): potassium (K_2_O) was 2:1:2. Phosphate fertilizer was applied as basal fertilizer at one time, and potassium fertilizer was applied at the same amount before ploughing and jointing stage. Water management and pest control and other related cultivation measures are implemented in accordance with the requirements of high yield cultivation.

Upon reaching maturity, rice was harvested, threshed, and air-dried until the moisture content stabilized at 14.5%. Dried rice grains were packed into net bags and stored in a constant-temperature and humidity-controlled incubator (HWS-250Y, Jinghong Experimental Equipment, Shanghai China) at a temperature of 25 ± 2°C and relative humidity of 50 ± 5% to simulate the storage conditions of a grain silo. Samples were collected at 0, 12, and 24 months (M) for analysis. 2,4,6-trimethylpyridine ≥(4,6-, GC grade) used as standard substance used as the internal standard for the determination of 2-AP content were purchased from Adamas Reagent (Switzerland). Dichloromethane (≥99.99%, GC grade) used for 2-AP extraction and GC–MS mobile phase was purchased from Adamas Reagent (Switzerland). All other reagents were of analytical grade.

### 2.2 Rice processing quality

Stored rice was analyzed to determine the brown rice rate (%), milled rice rate (%), and head rice ratio (%), as described by Xu (13), And calculate the F_HRR_(the percentage of the head rice ratio rate of rice at aging time *t* relative to that of 0 M rice),with three replicates performed for each measurement to ensure data reliability. The huller (SY88-TH, South Korea) and milling (Xiba, LTIM-2099, China) instruments were used for in this study. Calculate aligning to (Equations 1–3):


(1) $$\mathrm{BRR}=\frac{\text{brown rice quality}}{\text{rice quality}}\times 100\%$$

(2) $$\mathrm{MRR}=\frac{\text{milled rice quality}}{\text{rice quality}}\times 100\%$$

(3) $$\begin{array}{c} \mathrm{HRR}=\frac{\text{head rice quality}}{\text{unscreened rice}\;\text{quality}}\times \frac{\text{milled rice quality}}{\text{rice quality}}\times 100\% \end{array}$$

### 2.3 Determination of rice component content

Take the polished rice from 2.2 and grind it into powder using a grinder (A11, IKA, Germany), and total starch content (TSC) was determined using a total starch test box (DF-1-Y, Comin, Suzhou, China). As described by Tan et al. (14), Referring to the method of Kaufman (15), AC was measured using the iodine-binding method. Protein content (PC) was determined using a plant total nitrogen content test kit (PQD-1-G, Comin, Suzhou, China). Crude fat content (CFC) of rice was determined via Soxhlet extraction using the FOSS automatic Soxhlet extractor (SOXTEC2050, FOSS, Copenhagen, Denmark). Free fatty acid content (FFAC) was measured using an FFAC test kit (FFAZ-1-W, Comin, Suzhou, China). All measurements were repeated three times.

### 2.4 Fourier-transform infrared (FTIR) spectroscopy

As described by Shi et al. (16), structural changes in rice flour components during aging were analyzed via FTIR spectroscopy (FTIR Cary 610/670, Varian, Palo Alto, CA, USA). Rice flour was mixed with potassium bromide at a ratio of 1:100 and pressed into a pellet for FTIR analysis. The test wavelength range was 400–4,000 cm^−1^. Air was used as the background, with 64 scans for the background and 32 scans for each sample. Baseline correction, smoothing, and deconvolution were performed using the Resolutions Pro software.

### 2.5 X-ray diffraction (XRD)

According to the method described by Shi et al. (16) powder X-ray diffraction (D8 Advance, Bruker, Leipzig, Germany) was used to analyze the changes in crystal structure of rice during aging. The 2θ range was set from 5 to 35°, with a step length of 0.02° and a step speed of 0.6 s.

### 2.6 Rice color

Rice color was determined as described by Garber (17). Approximately 20 g of head rice was placed in a quartz dish, and L*, a*, b*, and transparency (Y) values of the samples were measured using a colorimeter (CM-5, Konicaminolta, Tokyo, Japan) under a standard D65 light source. All measurements were repeated three times.

### 2.7 Rice cooking characteristics

As described by Zhu (12), was used to determine the water absorption rate (WAR), expansion rate (ER), iodine blue value of rice soup (RS-IBV), dry matter content of rice soup (RSDM) and the pH value of rice soup. Weigh the whole polished rice, with the mass recorded as m, and measure the volume as v using the drainage method. Place it in a copper wire cage with a known mass of m_1_, wash it 5 times in flowing water, remove the rice bran, and then rinse it with distilled water once. Place it in a 200 mL tall beaker, add 50°C distilled water to 120 mL, and boil it in a boiling water pot for 20 min (starting at 100°C and heating it with a 2000 W electric furnace). Remove the copper wire cage and place it on the beaker without any rice soup dripping. Then place it on a clean dry gauze and cool it for 0.5 h. Weigh it as m_2_ and measure the volume v_1_ using the drainage method. After removing the copper wire cage, wait for the rice soup in the 200 mL tall beaker to cool to room temperature, and measure its pH value using a pH meter (PHSJ-3F, Yidian, Shanghai, China). Dilute the rice soup to 100 mL after measuring the pH value, centrifuge and take 10 mL into a small beaker with a known mass of m_3_. Dry and weigh it as m_4_. Take 1.0 mL of the centrifuged solution for measuring the dry matter of the rice soup and add it to about 50 mL of distilled water. Add 5 mL of 0.5 mol/L HCl solution and 0.2 g/100 mL iodine reagent to make up the volume. Measure the absorbance at 660 nm using a UV visible spectrophotometer (752, Shanghai Jinghua, China) and a 1 cm cuvette. Repeat the above steps three times and record them. Calculate aligning to (Equations 4–6):


(4) $$\begin{array}{c} \mathrm{WAR}=\frac{m}{m_{2}-m_{1}}\times 100\% \end{array}$$

(5) $$\begin{array}{c} \mathrm{ER}=\frac{v_{1}}{v}\times 100\% \end{array}$$

(6) $$\begin{array}{c} \text{RSDM}=\frac{m_{4}-m_{3}}{m}\times 100\% \end{array}$$

### 2.8 Rice taste quality

As described by Ma (18), appearance, hardness, stickiness, balance, and taste of cooked rice were evaluated using a taste analyzer (STA1A, Satake Corporation, Japan). Briefly, 30 g of head rice was washed thrice and soaked in a stainless- steel basin at a water-to-rice ratio of 1:1.3 for 30 min. Subsequently, the rice was cooked in an electric rice cooker (JT783, Midea, Shunde, China) for 30 min and kept warm for 10 min. After cooling for 20 min using an air-cooling device, it was further cooled at room temperature (25°C) for 90 min. For each sample, three rice cakes were prepared, and both sides of each cake were measured. According to the data obtained, F_TV_(the percentage of eating quality of rice aged at time t compared with that of 0 M rice.) is calculated.

### 2.9 Rice texture characteristics

As described by Wang (19), hardness (Hd), elasticity, stickiness (Stick), and equilibrium of rice were determined using a texture analyzer (TA. Xt. Plus, Stable Micro Systems, UK) equipped with a P/36R probe. The sample preparation method was the same as that described in section 2.7.

### 2.10 Determination of 2-acetyl-1-pyrroline (2-AP) content

As described by Yang (20), brown rice was ground into rice flour, and 2-AP content was measured via gas chromatography–mass spectrometry (8890-5977B, Agilent, USA). The chromatographic conditions are described below. HP-5MS (30 m × 250 *μ* × 0.25 μm) was used as the chromatographic column. Column temperature was initially maintained at 40°C for 1 min, raised to 65°C at a rate of 2°C/min, held for 1 min, and further increased to 250°C at a rate of 10°C/min. High-purity helium (purity > 99.999%) was used as the carrier gas at a flow rate of 1.20 mL/min. Constant pressure splitless injection was used at an injection volume of 1 μL. Mass spectrometry conditions were as follows: Electron impact ion source, with ion source temperature of 230°C, interface temperature of 250°C, quadrupole temperature of 150°C, and SIM scanning mode. Numbers of scanning ions were 79, 83, 111, and 121, respectively. These measurements were repeated three times.

### 2.11 Data processing and analysis

Data processing was performed using Excel 2019. Statistical analyses were conducted using the SPSS version 20.0 software. Graphs were plotted using the Origin 2021 software.

## 3 Results and discussion

### 3.1 Changes in the rice component contents during aging

**Figure 1:**
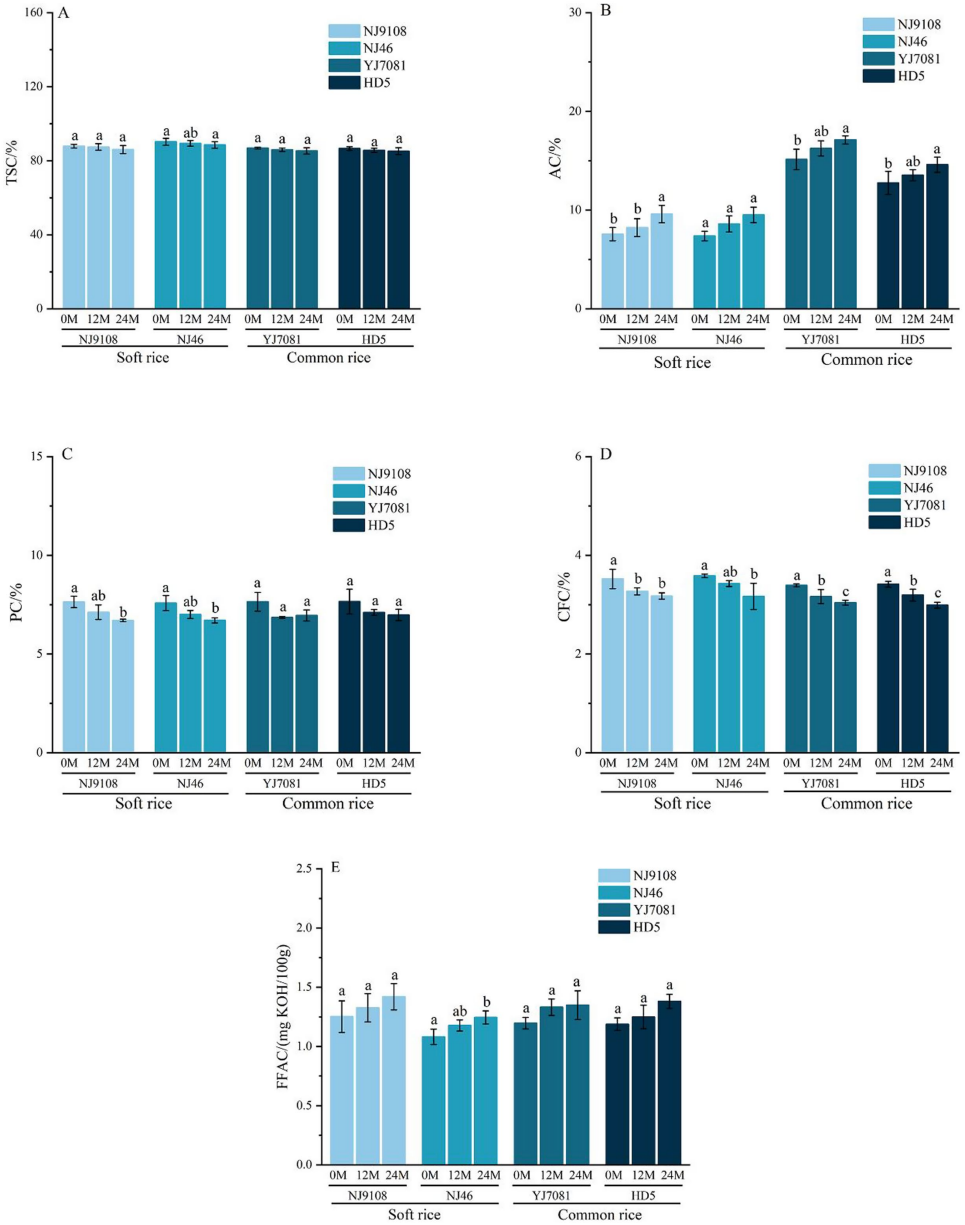
Changes in the rice component contents during aging.

Starch, proteins, and lipids are the primary rice constituents. During aging, variations in the contents and structures of these components serve as critical factors affecting the rice quality (21). As illustrated in Figures 1A–E, during rice aging, TSC, PC, and CFC gradually decreased, whereas AC and FFAC gradually increased with increasing storage time. After aging for 24 M, TSC of rice was decreased with an average reduction of 1.98% in the soft rice varieties and 1.72% in the common rice varieties. Changes in rice AC and TSC were inversely proportional. After aging for 24 M, average increase in AC was 2.09% in the soft rice varieties and 1.91% in the common rice varieties. AC was significantly higher in the common rice varieties than in the soft rice varieties. After aging for 24 M, average PC decreased by 0.91% in the soft rice varieties and 0.69% in the common rice varieties; however, the changes were not statistically significant. As shown in Figures 1D,E, CFC of rice gradually decreased, whereas FFAC gradually increased with increasing storage duration. After aging for 24 M, CFC values decreased by 0.38 and 0.39%, whereas FFAC values increased by 1.68 and 1.73 mg KOH/100 g in the soft and common rice varieties, respectively.

During rice aging, TSC, PC, CFC, AC, and FFAC underwent continuous changes, resulting in the deterioration of rice quality. With increasing aging duration, TSC and PC decreased, whereas AC increased in the soft rice and common rice varieties. This was possibly because of the gradual degradation of starch and proteins under the influence of relevant enzymes and partial conversion of amylopectin into amylose in the starch structure (22). High AC of rice grains increases the hardness of cooked rice (23), thereby decreasing its eating quality. In this study, average rate of increase in AC during aging was higher in the soft rice varieties than in the common rice varieties. Fatty acid content of rice is a critical indicator of its freshness. In this study, as aging progressed, CFC decreased, whereas FFAC increased in the soft and common rice varieties. This was possibly because of the hydrolysis reactions occurring during aging, leading to the formation of substances, such as peroxides, glycerol, and free fatty acids (24).

### 3.2 Changes in the rice component structures during aging

**Figure 2:**
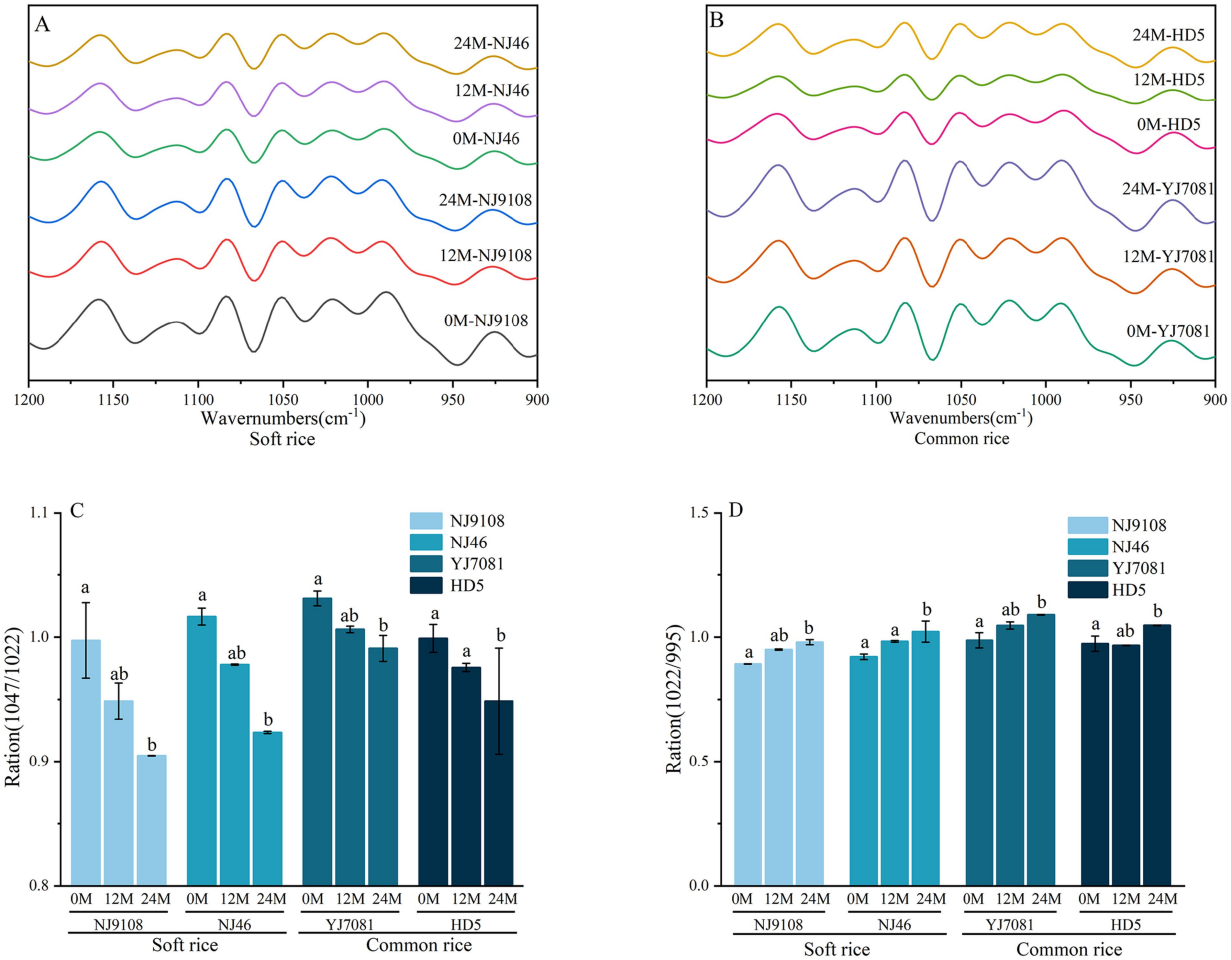
Changes in the rice starch structure during aging.

The FT-IR was used to record the spectra in the range of 1,200–900 cm^−1^ for evaluating starch short-range ordered structure. Figures 2A,B shows the deconvoluted FTIR spectra of rice starches. The intensity at 1047 cm^−1^and 1,022 cm^−1^ are, respectively, sensitive to changes in starch ordered and amorphous regions. Accordingly, the ratio of 1,047 cm^−1^ and 1,022 cm^−1^ band intensities (*R_1047/1022_*) to the ratio of 1,022 cm^−1^ and 955 cm^−1^ band intensities (*R_1022/955_*) was used to evaluate the short-range ordered structural changes of starch during aging in rice (25). Previous studies have confirmed that the 1,600 cm^−1^-1700 cm^−1^ region in FTIR spectra can be used to characterize the secondary structure of protein (26). Absorption peak in the range of 1,646–1,664 cm^−1^ corresponds to the *α*-helix structure of proteins. Absorption peaks within the ranges of 1,615–1,637 and 1,682–1,700 cm^−1^ are associated with the *β*-sheet structure of proteins. Absorption peak within the 1,664–1,681 cm^−1^ range indicates the β-turn structure of proteins (18), whereas that within the 1,637–1,645 cm^−1^ range reflects the random coil structure of proteins (26). In this study, FTIR was used to analyze the structural changes in rice components, such as starch and protein, during aging.

As illustrated in Figure 2, R*_1047/1022_* ratio of rice significantly decreased, whereas R*_1022/955_* ratio significantly increased with increasing aging duration. After aging for 24 M, average R*_1047/1022_* ratio decreased by 0.0927, whereas average R*_1022/955_* ratio increased by 0.0940 in the soft rice varieties. In contrast, average R*_1047/1022_* ratio decreased by 0.0447, whereas average R*_1022/955_* ratio increased by 0.8780 in the common rice varieties. These results indicated that the ordered structure of rice starch decreased during aging. Such changes occur because enzymes involved in starch metabolism degrade the helical structure within starch during aging (27), thereby causing dissociation of its aggregated structure and gradual degradation of starch. Short-range ordered structure of soft rice varieties declines faster than that of the common rice varieties. This difference affects the gelatinization and retrogradation characteristics of rice starch, leading to differences in the cooking and eating qualities of rice.

**Figure 3:**
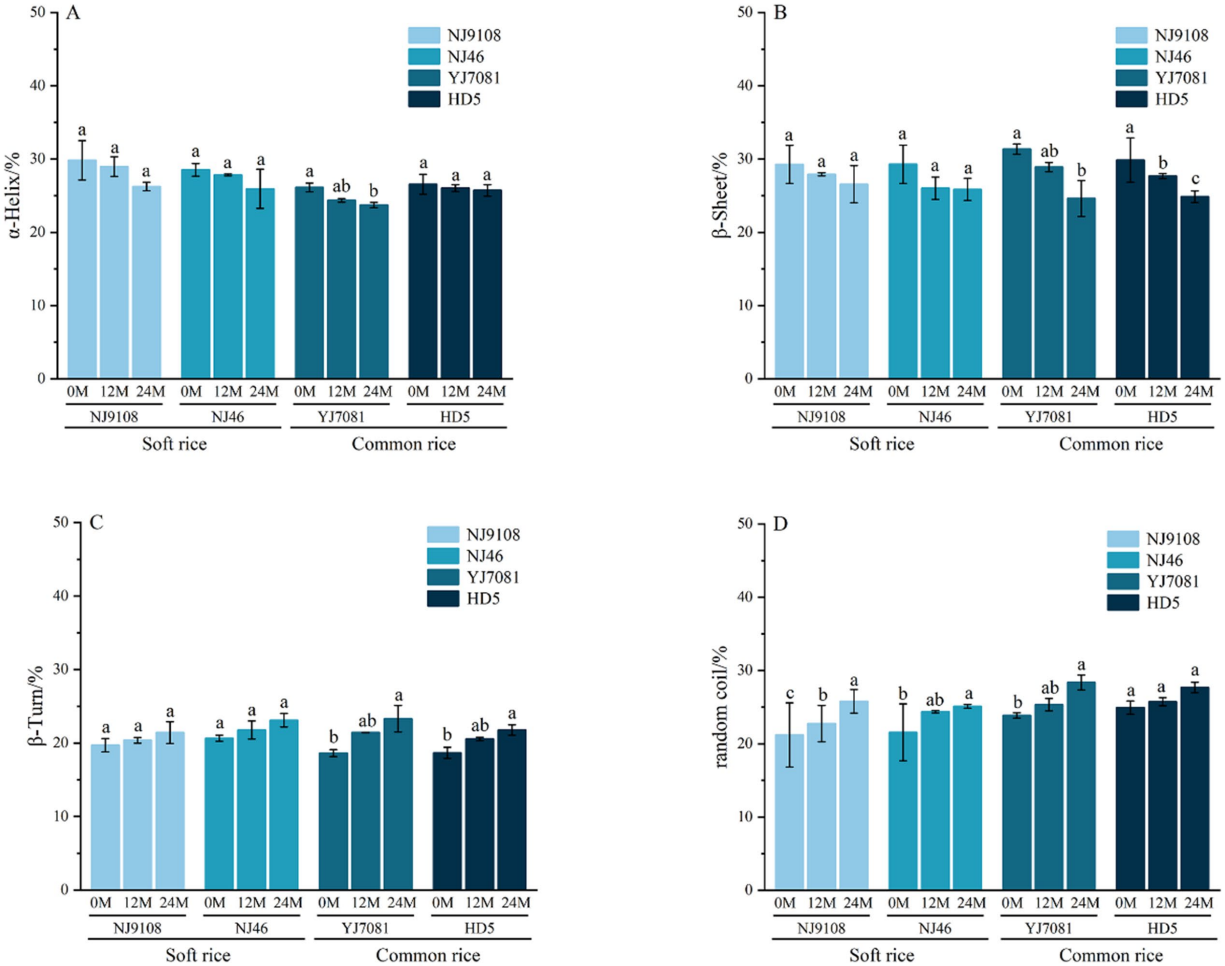
Changes in the rice protein secondary structure during aging.

As shown in Figure 3, proportions of *β*-sheet and *α*-helix structures in rice protein gradually decreased, whereas those of random coils and *β*-turns gradually increased with increasing aging duration. Notably, β-sheet was the predominant secondary structure in rice protein, consistent with previous reports (28). As illustrated in Figure 3B after aging for 24 M, average *β*-sheet content decreased by 3.07% in the soft rice varieties and 5.87% in the common rice varieties. These findings suggest that the protein secondary structure undergoes alterations during aging characterized by a reduction in protein aggregation and conversion of some β-sheet structures into β-turns. Consequently, protein molecules transition from a more ordered arrangement to a disordered state. Hydrophobic surface layer of β-sheet impacts the fluidity of water (29). In contrast to the common rice varieties, soft rice varieties showed a slower decline in β-sheet content in this study. This disparity influences the surface hydrophobicity of rice protein molecules, affecting the hardness of cooked rice and contributing to the differences in the textural properties of rice.

### 3.3 Changes in the relative crystallinity of rice starch during the aging

**Figure 4:**
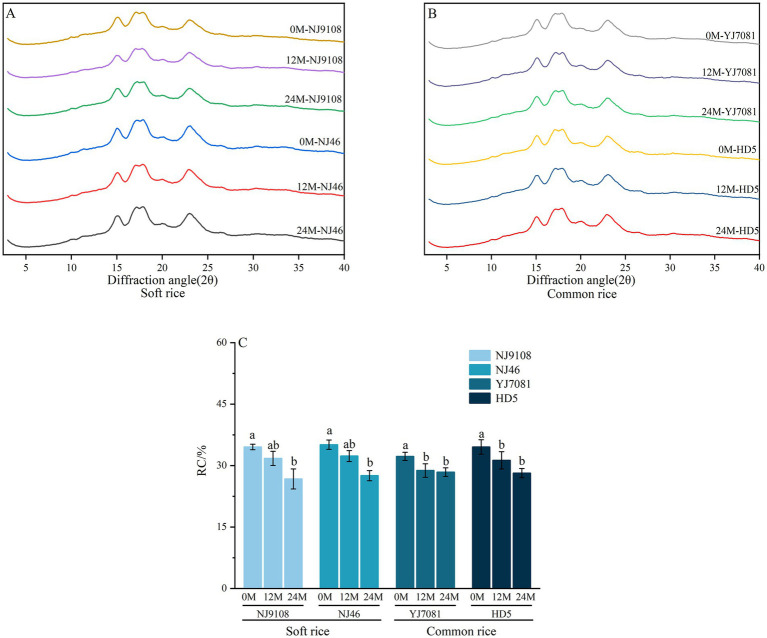
Changes in the relative crystallinity of rice starch during the aging.

As a typical semi-crystalline biopolymer, rice starch is composed of highly ordered crystalline regions and loosely disordered amorphous regions (30). The formation of the crystalline region is derived from the double helix structure formed by the self-assembly of starch chains (amylose and amylopectin) through hydrogen bonds and van der Waals forces. These helixes are further stacked to form layered crystals, and their long-range order can be quantitatively characterized by X-ray diffraction (XRD) (31). The changes of XRD pattern and starch crystallinity during rice aging are shown in Figure 4. All rice starches showed typical A-type crystal structure, and the characteristic diffraction peaks were located at 15 °, 17 °, 18 °, and 23 °, and the bi-modal signal of 17 ° and 18 ° was the most significant.

The relative crystallinity (RC%) of rice starch was further calculated. The results are shown in Figure 4C. As the aging time increases, the relative crystallinity of rice starch gradually decreases. Compared with common rice varieties, the relative crystallinity of soft rice varieties changed faster. After aging for 24 M, the relative crystallinity of soft rice starch decreased by 7.67% on average, and the relative crystallinity of common rice starch decreased by 5.11% on average. This decrease may be due to the enhanced activity of amylase during rice storage, resulting in loose packing of the lattice, destruction of the double helix structure and / or a shift in the double helix direction (27). In summary, with the extension of storage time, the crystal type of rice starch itself will not change, but it will affect the change of relative crystallinity of rice starch during aging.

### 3.4 Changes in the rice processing quality during aging

**Figure 5:**
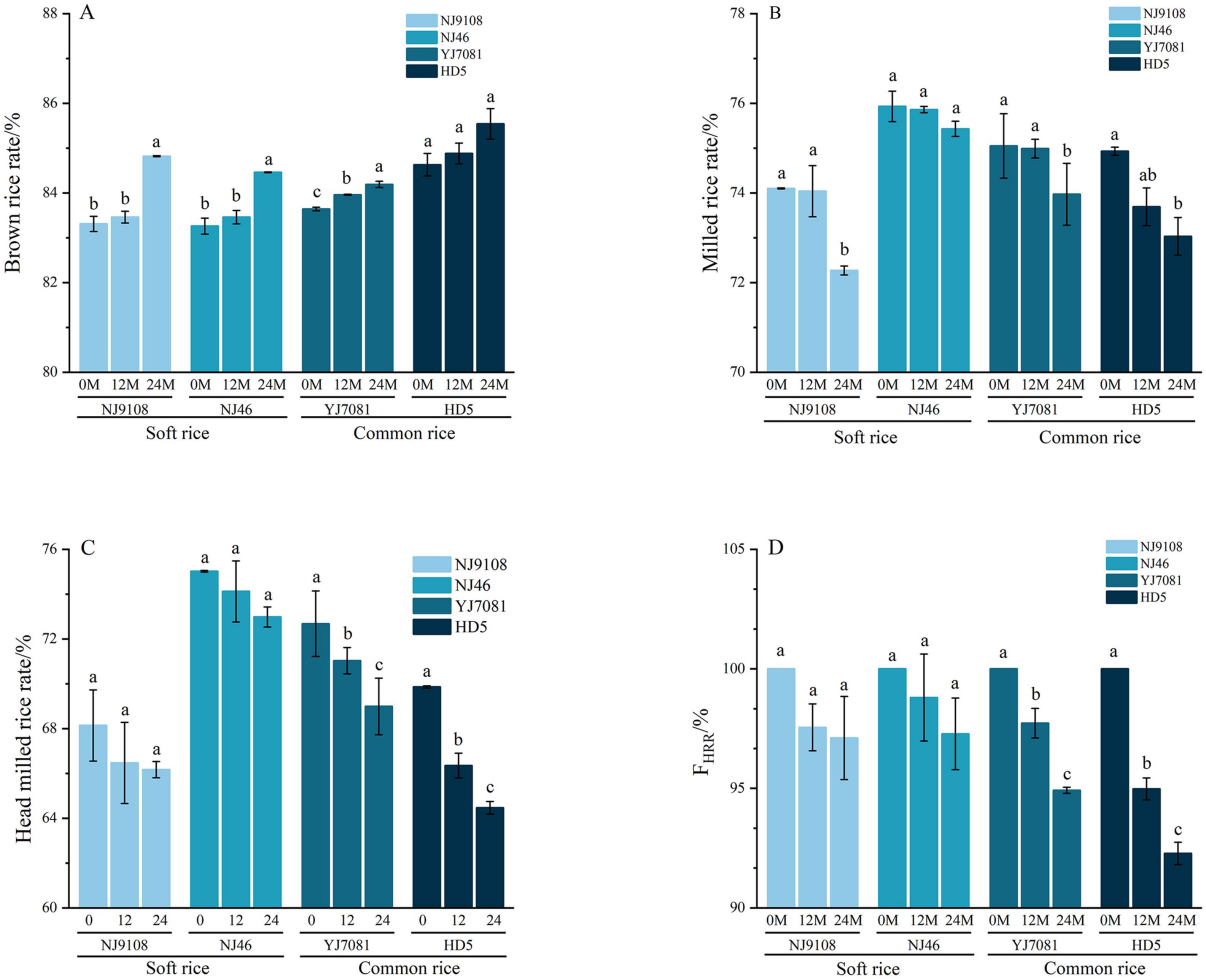
Changes in the rice processing quality during aging.

Processing quality constitutes a critical indicator in the rice quality evaluation system, encompassing brown rice rate, milled rice rate, and head rice ratio rate. During the aging, variations in processing quality can influence the yield of polished white rice as well as economic benefits (32). In this study, F_HRR_ is defined as the percentage of the head rice ratio rate of rice at aging time t relative to that of 0 M rice. Changes in the rice processing quality during aging are illustrated in Figure 5.

As shown in Figures 5A,B, brown rice rate increased, whereas milled rice rate decreased with increasing aging duration. As illustrated in Figures 5C,D, head rice ratio decreased with increasing aging duration. Notably, rate of decline during the 0–12 M aging period was significantly higher than that during the 12–24 M period. Specifically, head rice ratio decreased by 1.2–5.02%, with an average reduction of 2.74%, during the 0–12 M period, and 0.44–2.81%, with an average reduction of 1.86%, during the 12–24 M period. After 24 M of storage, head rice ratios of soft rice varieties decreased by 1.97 and 2.04%, respectively, whereas those of common rice varieties decreased by 3.69 and 5.39%, respectively. During the 0–24 M storage period, decline in the head rice ratio of soft rice varieties remained relatively stable, with no significant changes. In contrast, head rice ratio of the common rice varieties significantly decreased during the same period.

Aging duration significantly impacts the whole and milled rice yields (33), consistent with our findings. After aging for 24 M, brown rice rate increased, whereas milled rice rate and head rice ratio rates decreased in this study. This may be related to the change of AC, which influences the interactions between the starch molecules and other components, such as proteins and fats, in the rice kernel. These interactions lead to tight structural conformations (34), thereby affecting the hardness of rice and leads to the deterioration of rice processing quality.

### 3.5 Changes in rice appearance during aging

**Figure 6:**
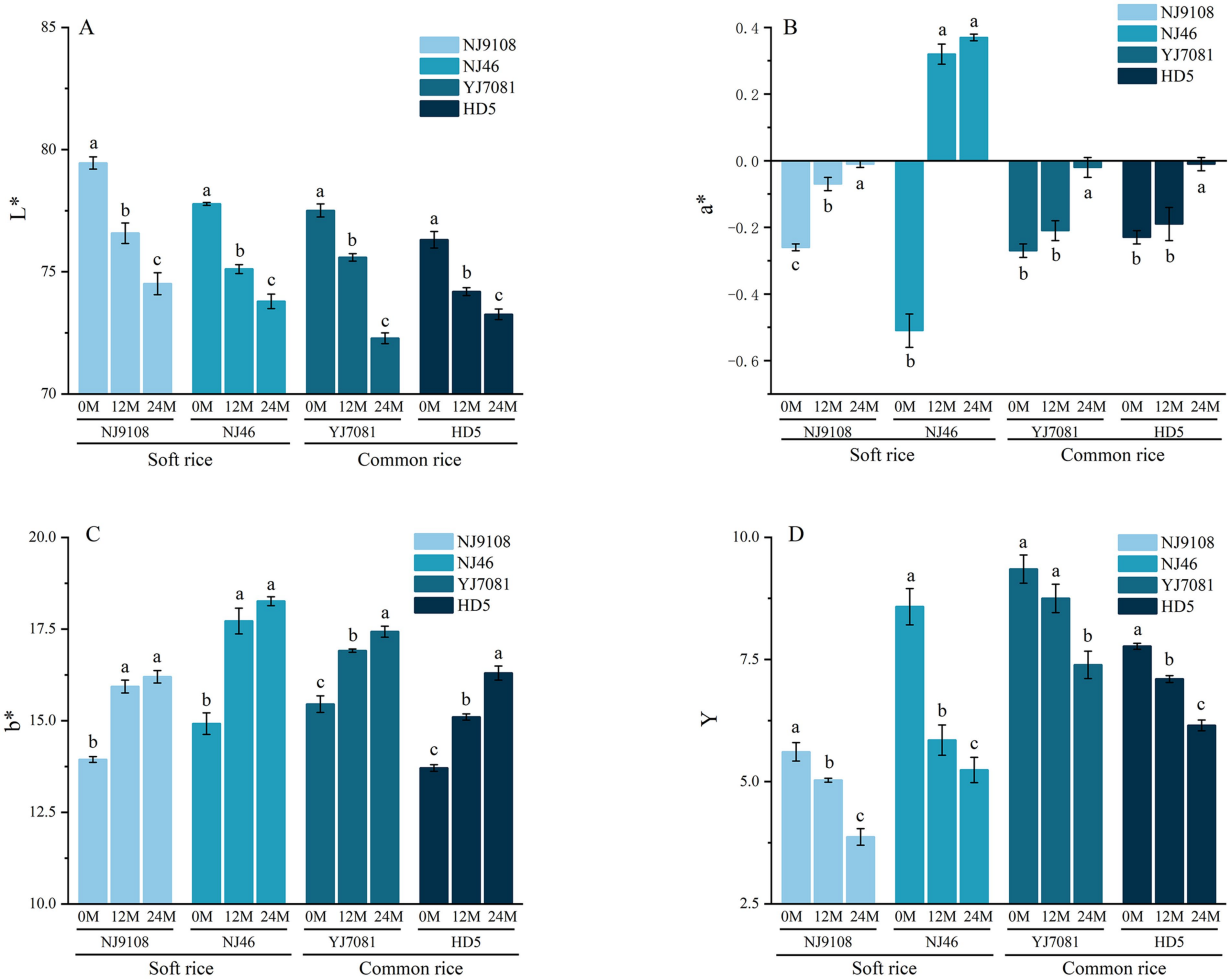
Changes in the rice processing quality during aging.

Rice appearance primarily encompasses its color, transparency, and chalkiness, which can be quantitatively analyzed using a colorimeter. As shown in Figures 6A–D, during rice aging, L* and Y values markedly decreased, whereas a* and b* values increased with increasing aging duration. During the 0–12 M aging period, rates of increase in a* and b* values were significantly higher in the soft rice varieties than in the common rice varieties. Specifically, a* and b* values of soft rice increased by 0.51 and 2.40, respectively, whereas those of common rice increased by 0.05 and 1.43, respectively. Moreover, rates of increase in a* and b* values gradually decreased in soft rice during the 12–24 M aging period. Specifically, a* and b* values of soft rice increased by 0.06 and 0.41, respectively, whereas those of common rice increased by 0.41 and 0.86, respectively.

The above-mentioned findings suggest that rice appearance undergoes significant changes during aging. Here, L* and Y values of rice decreased with increasing aging duration. During aging, rice color gradually darkens, brightness diminishes, and surface gloss decreases, possibly due to the reduced reflection of light (35). Increase in a* and b* values of rice is possibly because of the oxidation and Maillard reactions of lipids and other substances on the rice surface during storage (36). The observed decrease in CFC and increase in FFAC in this study confirmed the occurrence of these reactions, which resulted in the deterioration of rice appearance characterized by the gradual reddening and yellowing of rice grains. Compared to common rice, soft rice appeared duller and more yellow in color during the 0–12 M aging period. Its rate of quality decline became slower during the 12–24 M period.

### 3.6 Changes in the rice cooking characteristics during aging

Cooking characteristics of rice mainly include WAR and ER during cooking, along with the pH of the rice soup, RS-IBV, and RSDM. Cooking characteristics of rice are closely related to its cooking and eating qualities (37).

**Figure 7:**
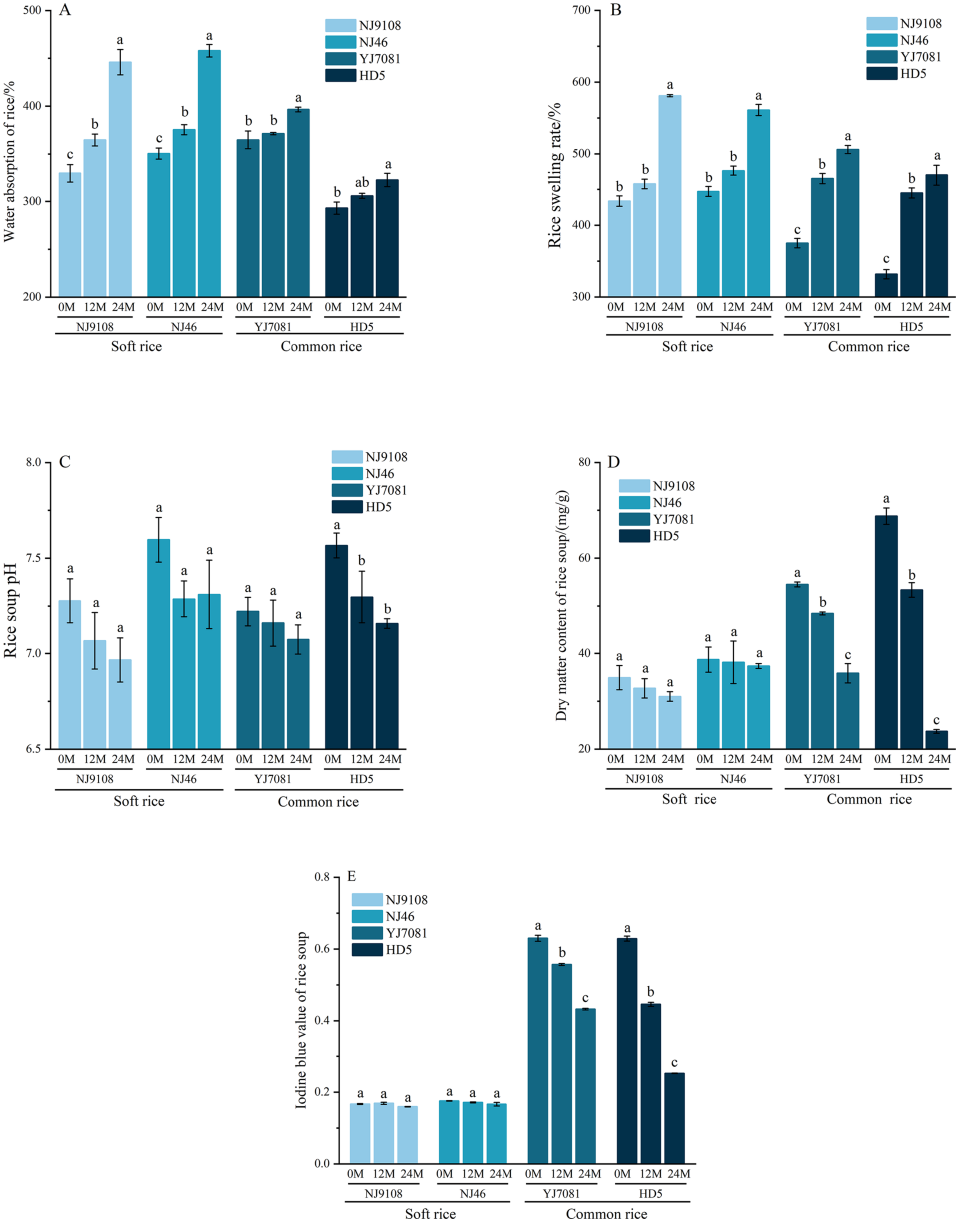
Changes in the rice cooking characteristics during aging.

As illustrated in Figures 7A–C, during aging, WAR and ER of rice significantly increased, whereas pH of rice soup decreased with increasing aging; however, this change was not statistically significant. As shown in Figures 7D,E, RSDM and RS-IBV decreased in the soft rice varieties and significantly decreased in the common rice varieties. During the 0–12 M aging period, WAR of rice increased by 1.79–10.63%, with an average increase of 5.98%. During the 12–24 M aging period, WAR of rice increased by 5.69–23.56%, with an average increase of 12.87%. Notably, WAR of soft rice varieties was higher than that of common rice varieties. During the 0–12 M aging period, ER of rice increased by 5.50–34%, with an average increase of 17.51%. During the 12–24 M aging period, ER of rice increased by 7.53–28.47%, with an average increase of 17.87%. Overall, soft rice varieties exhibited higher WAR and ER values than the common rice varieties. During the 0–24 M aging period, RSDM decreased by 3.51–65.52%, with an average decrease of 28.87%. Additionally, RS-IBV decreased by 4.49–59.86%, with an average decrease of 25.21%. Importantly, soft rice varieties exhibited lower RSDM and RS-IBV than the common rice varieties.

Rice WAR, ER, RSDM, and RS-IBV changed significantly, whereas pH value showed no obvious changes during aging. Increase in WAR and ER of rice during aging was possibly because of the degradation of cell walls containing proteins and other substances, which enhances the water absorption capacity of cells (38). After aging, WAR and ER of the common rice varieties were significantly lower than those of the soft rice varieties. This was possibly because of the stronger interaction between starch and protein molecules in the common rice varieties than in the soft rice varieties, leading to the formation of a more intact gel structure during retrogradation, which restricts water molecule migration (39). This increases the cooking time and hardness and reduces the palatability of common rice varieties compared to those of soft rice varieties. Consequently, RSDM and RS-IBV of soft rice varieties were lower than those of common rice varieties. This suggests that during the cooking process, soft rice varieties exhibit reduced amylose leaching. Cooked soft rice varieties exhibit a softer and stickier texture and better palatability than the cooked common rice varieties (40). Here, pH of the rice soup decreased during aging, possibly due to the progressive degradation of lipids over time, after aging for 24 months, the average soft rice CFC decreased by 0.38%. resulting in the production of various organic acids, including free fatty acids, after aging for 24 months, the average increase in soft rice FFAC was 1.68 mg KOH/100 g. Compared to the common rice varieties, soft rice varieties exhibited a faster rate of pH change, possibly because of their higher fat content that resulted in the generation of several acidic substances during aging.

### 3.7 Changes in the rice taste quality during aging

Eating quality of rice refers to the hardness, viscosity, appearance, taste, and texture of cold rice during consumption. Changes in the eating quality of rice during aging constitute an important index to assess its storage quality. Rice palatability meters are typically used to determine the palatability and eating quality of cooked rice (41). In this study, the F_TV_ was defined as the percentage of eating quality of rice aged at time t compared with that of 0 M rice.

**Figure 8:**
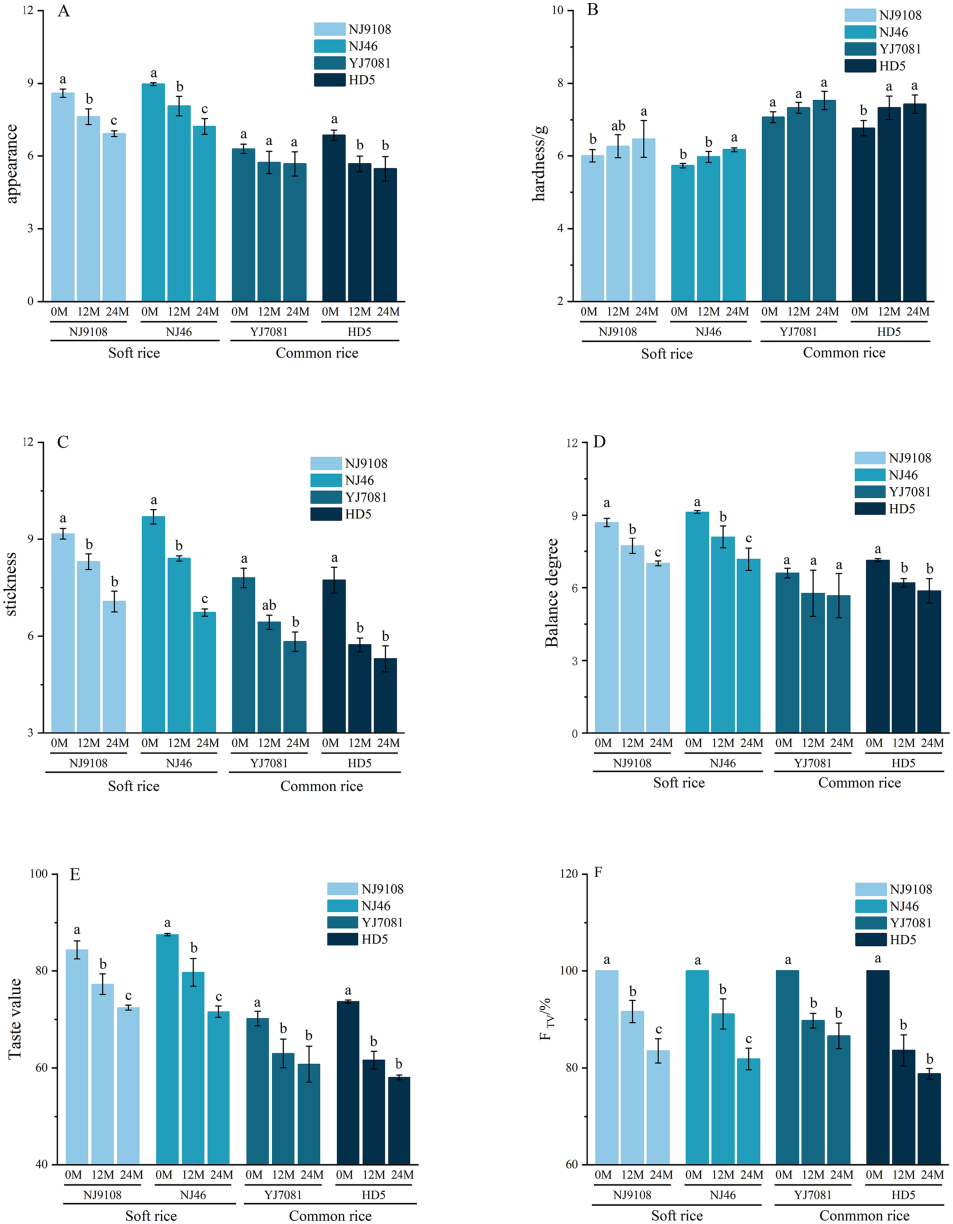
Changes in the rice taste quality during aging.

As shown in Figure 8, rice appearance, stickiness, taste value, and balance decrease with increasing aging duration, hardness of cooked rice increase with increasing aging duration. During the 0–12 M aging period, rate of decrease in cooked rice palatability was notably higher than that during the 12–24 M period. The range of decrease in cooked rice palatability was 9.37–16.48% (average = 11%) during the 0–12 M period and 3.13–9.16% (average = 6.33%) during the 12–24 M period. Notably, palatability of soft rice was significantly higher than that of common rice. Specifically, palatability values of soft rice varieties were 27.91, 32.34, and 23.16% higher than those of common rice varieties at 0, 12, and 24 M of aging, respectively. During the 0–24 M aging period, rate of decrease in palatability was considerably more stable in soft rice than in common rice. In the common rice varieties, rate of decrease in palatability was relatively rapid in the early aging stages and significantly reduced in the later aging stages. During the 0–12 M aging period, rate of decrease in palatability was markedly lower in the soft rice varieties than in the common rice varieties. In contrast, during the 12–24 M aging period, rate of decrease in palatability was significantly higher in the soft rice varieties than in the common rice varieties.

The above-mentioned results showed that the eating quality of rice markedly declined during aging. Notably, soft rice varieties exhibited a slower rate of eating quality decline or no significant difference in eating quality compared to the common rice varieties during the early aging stages. However, during the later aging stages, deterioration rate of eating quality was significantly higher in the soft rice varieties than in the common rice varieties. Importantly, eating quality of soft rice varieties remained significantly superior to that of common rice varieties throughout the aging period.

### 3.8 Changes in the textural properties of cooked rice during aging

Textural characteristics of rice, including its hardness, stickiness, elasticity, and balance (42), are used to assess the palatability of cooked rice during consumption.

**Figure 9:**
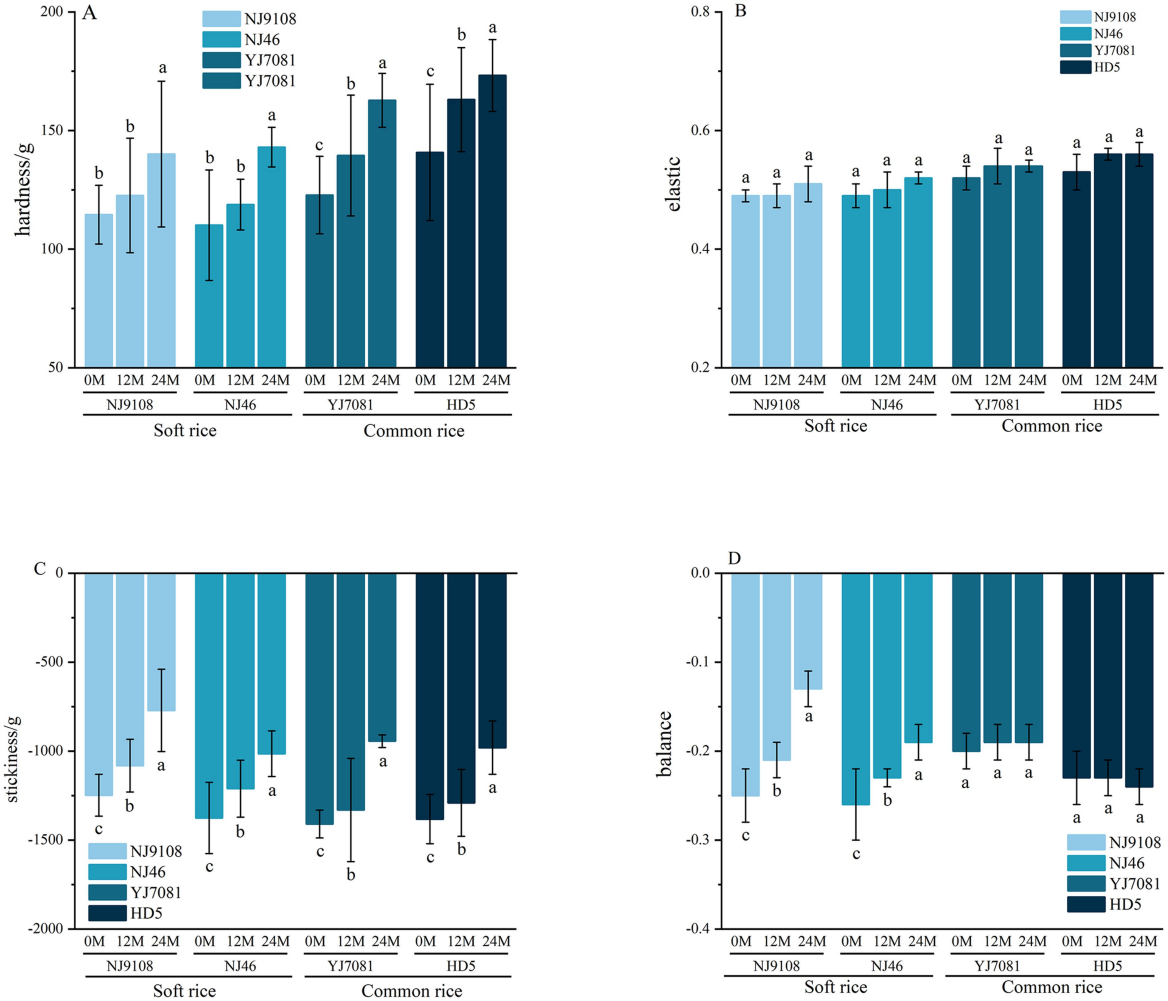
Changes in the rice texture during aging.

Figure 9 shows that the hardness and elasticity of rice and cooked rice increased, whereas stickiness and balance decreased with increasing aging duration. In contrast, hardness of the cooked soft rice varieties was significantly lower than that of the cooked common rice varieties. Hardness values of the cooked soft rice varieties were 19.44, 20.20, and 26.43 g lower than those of the common rice varieties at 0, 12, and 24 M of aging, respectively. Hardness of the cooked soft rice varieties increased by 26.09%, whereas that of the common rice varieties increased by 27.79% after 24 M of aging. Elasticity of the cooked soft rice varieties increased by 4.59%, whereas that of the common rice varieties increased by 5.23% after 24 M of aging. Stickiness of the cooked soft rice varieties decreased by 32.22%, whereas that of the common rice varieties decreased by 31.03% after 24 M of aging. Notably, increase in equilibrium was significantly higher in soft rice than in common rice. Specifically, equilibrium of the cooked soft rice varieties increased by 37.86%, whereas that of the cooked common rice varieties increased by only 1.3% after 24 M of aging.

Textural properties of rice underwent significant changes during aging. Specifically, hardness markedly increased, whereas stickiness notably decreased during aging. Additionally, equilibrium of soft rice increased substantially during aging. Overall, hardness was significantly higher in the common rice varieties than in the soft rice varieties both before and after aging. Furthermore, after 24 M of aging, absolute value of stickiness was significantly higher in the soft rice varieties than in the common rice varieties. These results further confirm that the eating quality of soft rice is superior to that of common rice during aging.

### 3.9 Changes in the rice aroma quality during aging

Aroma quality is a critical attribute of rice quality, with consumers preferring rice varieties with a high aroma quality. Notably, 2-AP, characterized by its popcorn-like fragrance, is the primary constituent of rice aroma, acting as a key indicator for distinguishing fragrant rice from non-fragrant rice. Its concentration is commonly used as an indicator of the rice aroma quality. In this study, F 2-AP was defined as the ratio of the 2-AP content in rice aged for t M to that in rice aged for 0 M.

**Figure 10:**
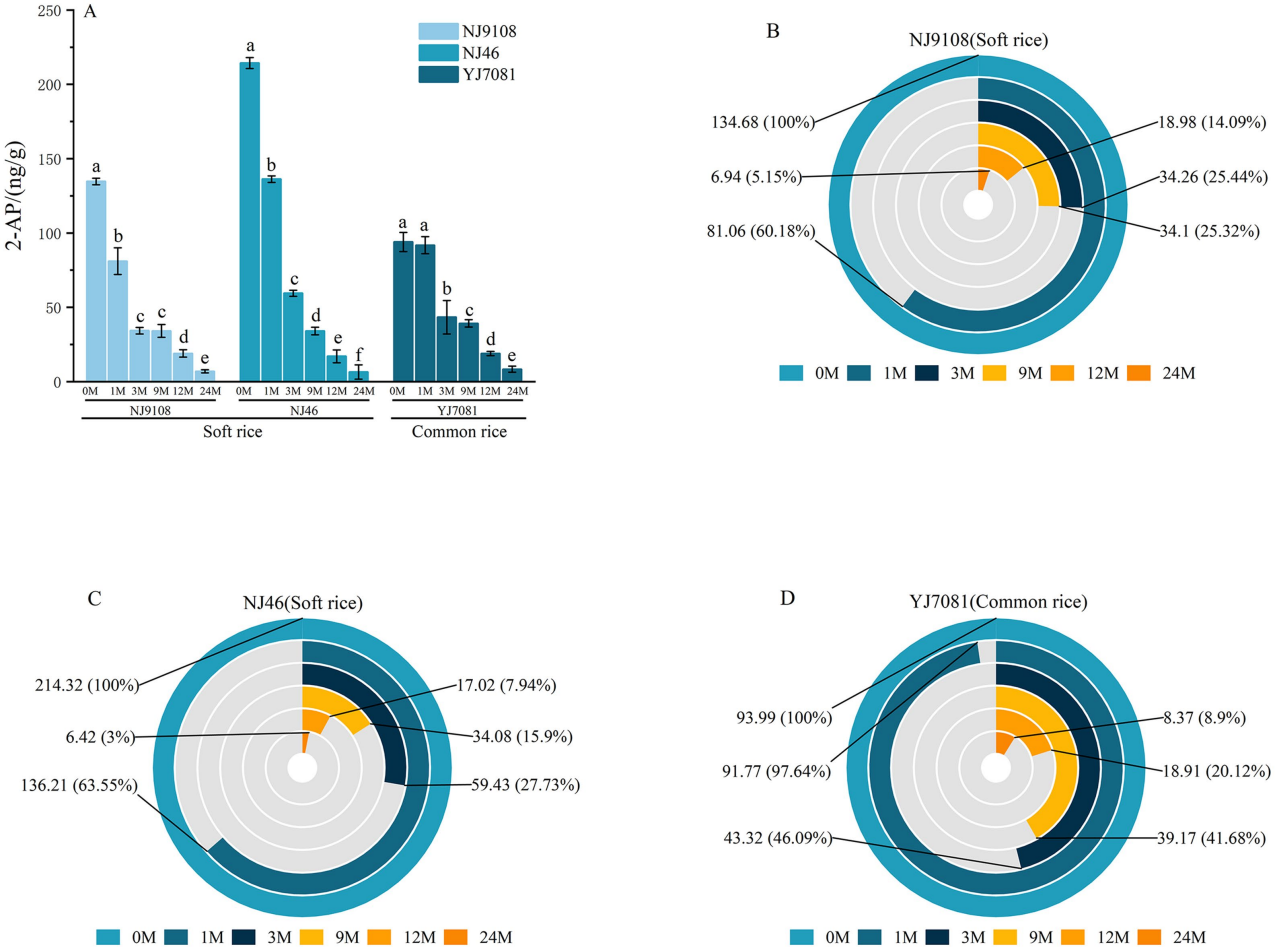
Changes in the rice aroma quality during aging.

The perception threshold of 2-AP in rice was 0.02 ng/L. As show in Figure 10, soft rice varieties NJ9108 and NJ46 and common rice variety YJ7081 were categorized as fragrant, whereas common rice variety HD5 was classified as non-fragrant. Specifically, 2-AP content decreased substantially with increasing aging time. Specifically, 2-AP content exhibited the most pronounced decrease during the 0–3 M aging period, with reductions of 73.42 and 53.91% in the soft and common rice varieties, respectively. After 24 M of aging, 2-AP content decreased by 95.93% in the soft rice varieties and 91.10% in the common rice varieties.

Rice aroma undergoes substantial deterioration during aging. Aroma quality deteriorates rapidly within the initial 3 M of aging, after which the deterioration rate progressively slows down with increasing aging duration. In this study, soft rice varieties exhibited significantly higher 2-AP content than the common rice varieties. Furthermore, rate of decrease in 2-AP content during aging was faster in the soft rice varieties than in the common rice varieties.

### 3.10 Correlation analysis of relevant indicators during rice aging

To elucidate the mechanisms underlying the quality changes during soft rice aging, we performed correlation analysis of the rice components, structure, and quality.

**Figure 11:**
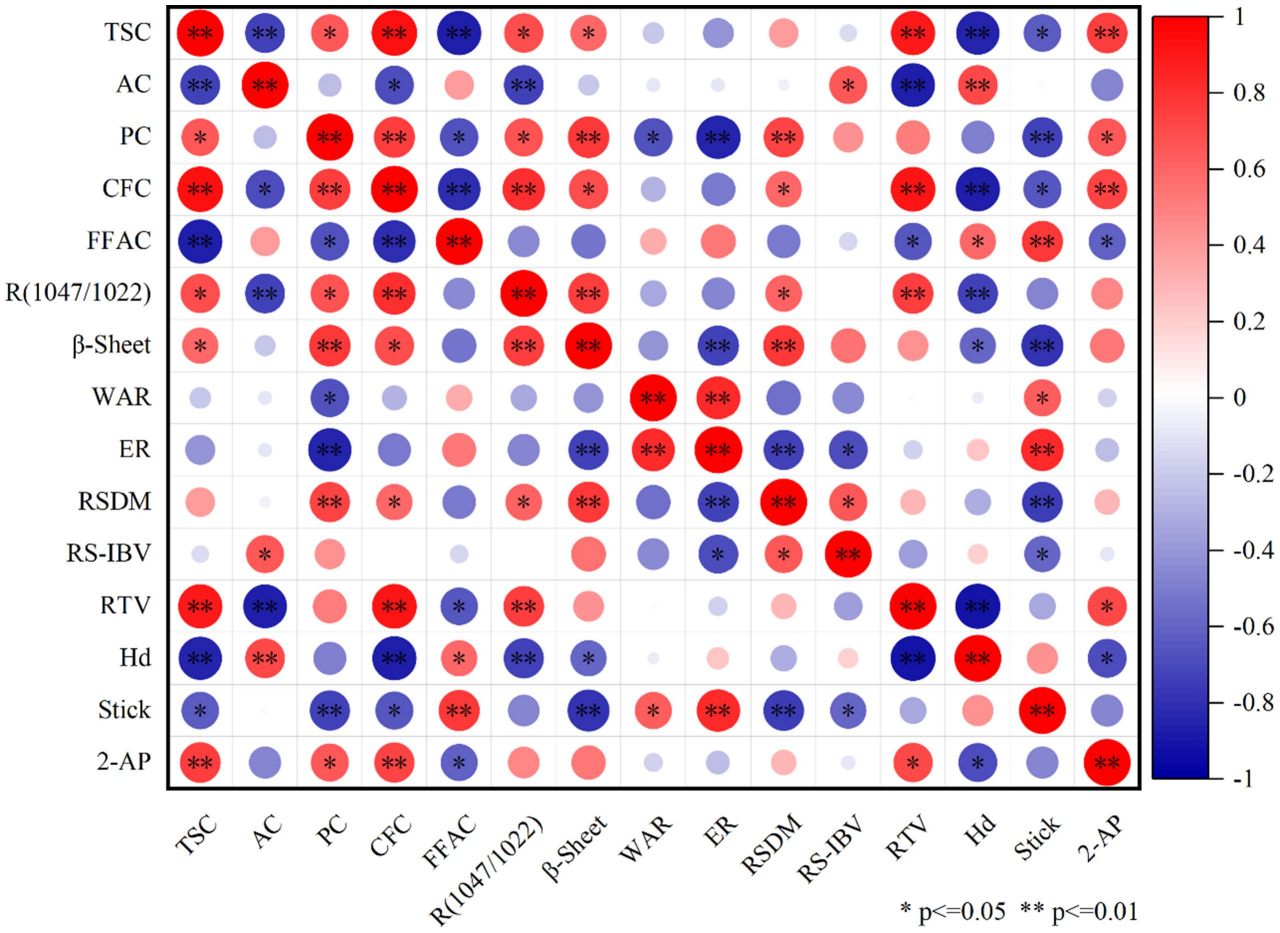
Correlation analysis of relevant indicators during rice aging. *p < 0.05, significant; **p < 0.01, highly significant.

As shown in Figure 11, eating quality of cooked rice exhibited significant positive correlations with TSC and R*_(1,047/1022)_* and significant negative correlations with AC and hardness. This was possibly because some linkage chains of amylopectin in rice starch are cleaved by debranching enzymes and converted into amylose, thereby increasing the AC during rice aging. During cooking, several amylose molecules form solid gel structures during retrogradation (43), thereby increasing the hardness and decreasing the eating quality of cooked rice. Notably, eating quality of soft rice varieties was significantly higher than that of common rice varieties throughout the aging period. This was possibly because of the considerably higher AC in the common rice varieties than in the soft rice varieties. AC in the common rice varieties further increased during aging, facilitating the interaction between the amylose molecules and long-side-chain amylopectin molecules, thereby promoting the formation of more stable double-helix structures (44). Consequently, the overall starch structure became less susceptible to disruption during cooking, leading to the inhibition of starch gelatinization and a subsequent reduction in the eating quality of the cooked rice. As depicted in Figure 2, despite the deterioration of the short-range ordered starch structure in the common rice varieties during aging, the rate of deterioration was relatively slow. This suggests that the starch structures of common rice varieties are more stable and exhibit higher resistance to gelatinization during cooking. Despite exhibiting superior eating quality, soft rice varieties showed a higher deterioration rate during storage than the common rice varieties.

Hardness of cooked rice is a critical factor influencing its eating quality (45). As shown in Figure 10, AC exhibited a highly significant positive correlation with the hardness of cooked rice. In contrast, R*_(1,047/1022)_* exhibited a highly significant negative correlation and *β*-sheet structure exhibited a significant negative correlation with the hardness of rice. Decrease in the β-sheet structure in rice during aging (Figure 3) suggests that aging degrades the secondary structure of rice proteins, resulting in the transformation of protein molecules from an ordered to a disordered structure. Furthermore, degree of transformation increased with the aging duration. During aging, *α*-helix and β-sheet structures unwind, weakening the protein molecular structure and increasing the number of disordered molecules. This increases the number of hydrophobic residues on the surface of protein molecules and strengthens the surface hydrophobicity (46), further increasing the hardness and eating quality deterioration of rice (47). As shown in Figure 3, β-sheet deterioration rate was lower in the soft rice varieties than in the common rice varieties. Consequently, increase in rice hardness during storage was smaller in soft rice than in common rice, leading to the superior eating quality of soft rice, even during aging.

Aroma component 2-AP of rice is extremely volatile and readily undergoes volatilization and dissipation. Additionally, interactions with rice components, such as proteins, starch, and fat, influence its dissipation (48). As shown in Figure 10, 2-AP content of rice exhibited highly significant positive correlations with its TSC and CFC, a significant positive correlation with its PC, and a significant negative correlation with its CFC. Rice starch exhibits a complex multiscale structure. The 2-AP molecules are adsorbed into starch granule pores (49). Amylose is prone to forming complexes with liposoluble 2-AP molecules (50), exerting an embedding effect on the 2-AP molecules. Rice proteins contain hydrophobic groups, which bind to the non-polar segments of 2-AP (51) and retain the aroma molecules. Therefore, reduction in TSC and PC (Figure 1), along with the modifications of starch and protein molecular structures, promotes the release of 2-AP molecules from rice during aging. The rate of decline in 2-AP content was faster in the soft rice varieties than in the common rice varieties. This was mainly attributed to the higher AC and more stable starch structure of common rice varieties than of soft rice varieties. Such structural characteristics support complexation with 2-AP molecules, thereby enhancing the stability and delaying the dissipation of 2-AP (48).

## 4 Conclusion

The research findings demonstrate that during the aging, the levels of total starch, fat, and protein in both soft rice and common rice progressively decrease, while the amylose content increases. Notably, the rate of increase in amylose content is higher in soft rice than in common rice. The short-range ordered structure of starch and the secondary structure of protein in both types of rice gradually degrade as the aging period progresses. In comparison to common rice, the degradation rate of the short-range ordered structure in soft rice occurs more rapidly. In addition, with the extension of aging time, the relative crystallinity of rice starch de-creased gradually. During the aging, the processing and appearance qualities of both soft and common rice deteriorate. The cooking and eating quality, as well as aroma quality, of soft rice are significantly superior to those of common rice. During the aging, the changes of component content and starch short-range ordered structure in soft rice were more obvious, leading to a greater deterioration rate in qualities such as cooking and eating, and aroma in soft rice compared to common rice.

## Data Availability

The original contributions presented in the study are included in the article/supplementary material, further inquiries can be directed to the corresponding author/s.
